# The landscape of global research on diabetic neuropathy

**DOI:** 10.3389/fendo.2023.1220896

**Published:** 2023-11-07

**Authors:** Mitra Tavakoli, Doris Klingelhöfer, Hassan Fadavi, David A. Groneberg

**Affiliations:** ^1^ Exeter Centre of Excellence for Diabetes Research, National Institute for Health and Care Research (NIHR), University of Exeter Medical School, Exeter, United Kingdom; ^2^ Institute of Occupational, Social and Environmental Medicine, Goethe University Frankfurt, Frankfurt, Germany

**Keywords:** diabetic neuropathy, peripheral neuropathy, research, impact, publication, networking, global research

## Abstract

**Introduction:**

Diabetic neuropathy (DN) is a prevalent and debilitating complication of diabetes, imposing a significant burden on individuals and healthcare systems worldwide. This study presents a comprehensive analysis of the global research landscape in DN, aiming to provide scientists, funders, and decision-makers with valuable insights into the current state of research and future directions.

**Methods:**

Through a systematic review of published articles, key trends in DN research, including epidemiology, diagnosis, treatment strategies, and gaps in knowledge, are identified and discussed.

**Results:**

The analysis reveals an increasing prevalence of DN alongside the rising incidence of diabetes, emphasizing the urgent need for effective prevention and management strategies. Furthermore, the study highlights the geographical imbalance in research activity, with a majority of studies originating from high-income countries.

**Discussion:**

This study underscores the importance of fostering international collaboration to address the global impact of DN. Key challenges and limitations in DN research are also discussed, including the need for standardized diagnostic criteria, reliable biomarkers, and innovative treatment approaches. By addressing these gaps, promoting collaboration, and increasing research funding, we can pave the way for advancements in DN research and ultimately improve the lives of individuals affected by this debilitating condition.

## Introduction

The global incidence of both type 1 diabetes (T1DM) and type 2 diabetes (T2DM) is on the rise among children, adolescents, and adults. The recent coronavirus (COVID-19) pandemic has further exacerbated the incidence of T2DM, creating a “tsunami” effect in the post-pandemic era (1). Additionally, there has been an observed increase in T1DM cases among children infected with COVID-19 (2).

The International Diabetes Federation (IDF) has highlighted the alarming global prevalence of type 1 and type 2 diabetes, with approximately half a billion individuals currently affected. This constitutes around 10.5% of the world’s adult population. Furthermore, IDF projects a substantial increase in the number of individuals living with diabetes, estimating a 25% rise by 2030 and a staggering 51% increase, reaching over 750 million individuals, by 2045. These projections emphasize the urgent need for effective prevention, management, and healthcare strategies to address the growing burden of diabetes on a global scale (3). Despite substantial advancements in clinical care, diabetes remains one of the leading causes of death worldwide, ranking within the top 10 (4).

In 2021, the prevalence of diabetes was found to be higher in high-income countries, with a rate of 11.1%, compared to low-income countries, where the rate was 5.5%. However, projections indicate that the most significant relative increase in diabetes prevalence between 2021 and 2045 is expected to occur in middle-income countries, with a rate of 21.1%. This increase surpasses that of both high-income countries, projected at 12.2%, and low-income countries, projected at 11.9%. Notably, countries undergoing economic transitions from low-income to middle-income status, particularly those in the Middle East and North Africa region, are expected to experience a substantial rise in diabetes prevalence (5). With the escalating prevalence of diabetes, it is anticipated that the burden of diabetes-related complications will also rise. In 2021, global expenditures related to diabetes healthcare were estimated to be around 966 billion USD. However, by 2045, these expenditures are projected to reach approximately 1,054 billion USD. This upward trend in healthcare expenditures underscores the growing economic impact of diabetes on a global scale (3).

Diabetic neuropathy (DN) is a prevalent, incapacitating, and economically burdensome chronic complication of diabetes, impacting up to 50% of patients and leading to significant disability and diminished quality of life. This poses substantial challenges for healthcare costs and society, particularly considering that these individuals are often in their prime careers and earning years when diabetes-related complications commonly arise.

Regrettably, our understanding of the epidemiology and natural history of diabetic neuropathy remains insufficient, partially attributed to inadequate patient selection and the inconsistent criteria employed in clinical trials and neuropathy studies. In its early stages, DN frequently lacks symptomatic manifestation; however, once symptoms and evident deficits appear, the irreversible nature of the condition becomes apparent. Consequently, timely diagnosis and intervention are crucial to forestall the development and progression of diabetic neuropathy.

The diagnosis of DN, ascertaining its global prevalence and incidence rates, presents substantial challenges. Diverse viewpoints exist concerning the efficacy of expanding screening initiatives to facilitate early diagnosis and initiation of treatment prior to disease onset and progression. Despite the progress made in research over time, DN continues to impose a significant burden on clinicians and healthcare systems worldwide due to the intricacies associated with diagnosis, the high cost of treatment, and the multidisciplinary approach necessary for effective management (6).

While sensitive biomarkers exist for other microvascular complications of diabetes, such as retinopathy and nephropathy, the absence of comparable biomarkers for diabetic neuropathy (DN) poses a significant challenge. Currently, there is a pressing need to identify reliable surrogate biomarkers that can effectively monitor the early neuropathic alterations in DN, thereby aiding in drug discovery and advancing therapeutic interventions.

The absence of sensitive biomarkers specific to DN underscores the unmet requirement for robust indicators that can accurately detect and track the onset and progression of neuropathic changes associated with the condition. Such biomarkers would not only enhance our understanding of the underlying mechanisms but also serve as valuable tools in evaluating the efficacy of potential therapeutic interventions.

Addressing this crucial gap in biomarker research for DN holds immense potential to facilitate early detection, monitor disease progression, and contribute to the development of novel treatment strategies. By identifying reliable surrogate biomarkers, researchers can accelerate the advancement of diagnostic and therapeutic approaches, ultimately improving the management and outcomes of patients affected by DN (6).

Regrettably, despite significant progress in managing diabetes and its complications, there is currently no specific treatment available for diabetic neuropathy. This lack of effective therapy is frustrating, as diabetic neuropathy continues to pose a significant challenge for patients. While there are treatments to manage symptoms and slow down progression, they primarily focus on symptomatic relief rather than addressing the underlying cause of the neuropathy. The complex nature of diabetic neuropathy, involving multiple factors and mechanisms, contributes to the difficulty in developing targeted therapies. However, ongoing research efforts aim to explore new pharmacological agents, neuroprotective strategies, and pathways involved in the condition, offering hope for the future development of effective treatments to alleviate the burden of diabetic neuropathy and enhance the lives of affected individuals (7).

Clinical and pre-clinical studies are crucial for addressing diabetic neuropathy as a devastating complication of diabetes. Successful clinical trials are needed to combat this condition and improve the lives of affected individuals. By conducting robust trials, researchers can enhance our understanding, explore novel treatments, and benefit patients. Raising global awareness of diabetic neuropathy drives attention, resources, and collaboration for this challenge. It fosters research funding, multidisciplinary collaborations, and innovative approaches for prevention, diagnosis, and treatment. Acknowledging the importance of these studies and conducting successful trials is vital for addressing the impact of diabetic neuropathy and improving affected individuals’ quality of life.

The primary objective of this study was to provide a comprehensive overview of global research on diabetic neuropathy, specifically focusing on publications in the field. By examining these publications, the authors aimed to determine the extent of existing research and shed light on the areas where knowledge gaps exist. The findings from this study serve multiple purposes, including identifying the specific areas that require further funding and research efforts to bridge these gaps in understanding. By elucidating the research landscape and identifying knowledge gaps, the authors hope to emphasize the necessity of tailored interventions and precision medicine strategies to effectively address the diverse manifestations and underlying mechanisms of DN.

## Methods and data source

The analyses presented in this study rely on the methodological approaches of the New Quality and Quantity Indices in Science (NewQIS) bibliometric platform. Established in 2009, NewQIS aims to offer a comprehensive examination of research output across various biomedical fields and topics. The platform employs a combination of established chronological and geographic evaluations alongside advanced parameters and visualization techniques. By integrating these methodologies, NewQIS provides an in-depth analysis of the research landscape in biomedical disciplines, enabling a robust assessment of scientific productivity and impact (8).

The platform constantly delivers new findings on a wide range of biomedical topics. The indices used are constantly being developed and adapted to the topics. In addition to absolute publication numbers, metrics are evaluated using socio-economic characterizations of the publishing countries, providing more intensive insight into the research landscape on scientific topics.

The default data source for all NewQIS analyses is the Web of Science Core Collection (WoS) database. WoS is one of the most established online scientific databases that provides all metadata for listed entries, including citation counts. It requires quality standards that are ensured by an impact factor for all listed journals and a properly conducted peer review process.

The search term used was elaborated using the Medical Subject Heading (MeSH) Thesaurus to include all synonymous terms for Diabetic Neuropathy. The time frame was set to the period from 1900 to 2021, and only original articles were included in the analysis. After retrieving the metadata from the WoS, the data were structured, standardized, and entered into an MS Access database for further analysis.

The analyses included absolute figures for articles, citations and countries, as well as relative values in the form of ratios of article numbers to socioeconomic values. The population size and economic power (Gross Domestic Product = GDP) of the countries were taken into account (9). Trends and development of research patterns were elaborated by annual values. Research priorities were identified by analyzing the original WoS categories and authors’ keywords. In addition, the most cited articles were identified.

Geographic results were partially visualized using density equalization map projections (DEMP) according to an algorithm developed by Gastner and Newman (10). This method distorts the size of countries depending on the parameter being analyzed. This results in increasing the size of countries with high values and decreasing the size of countries with low values. Keyword analyses were performed and visualized using van Eck and Waltman’s VOSviewer (11).

## Results

### General parameters and keywords

**Figure 1:**
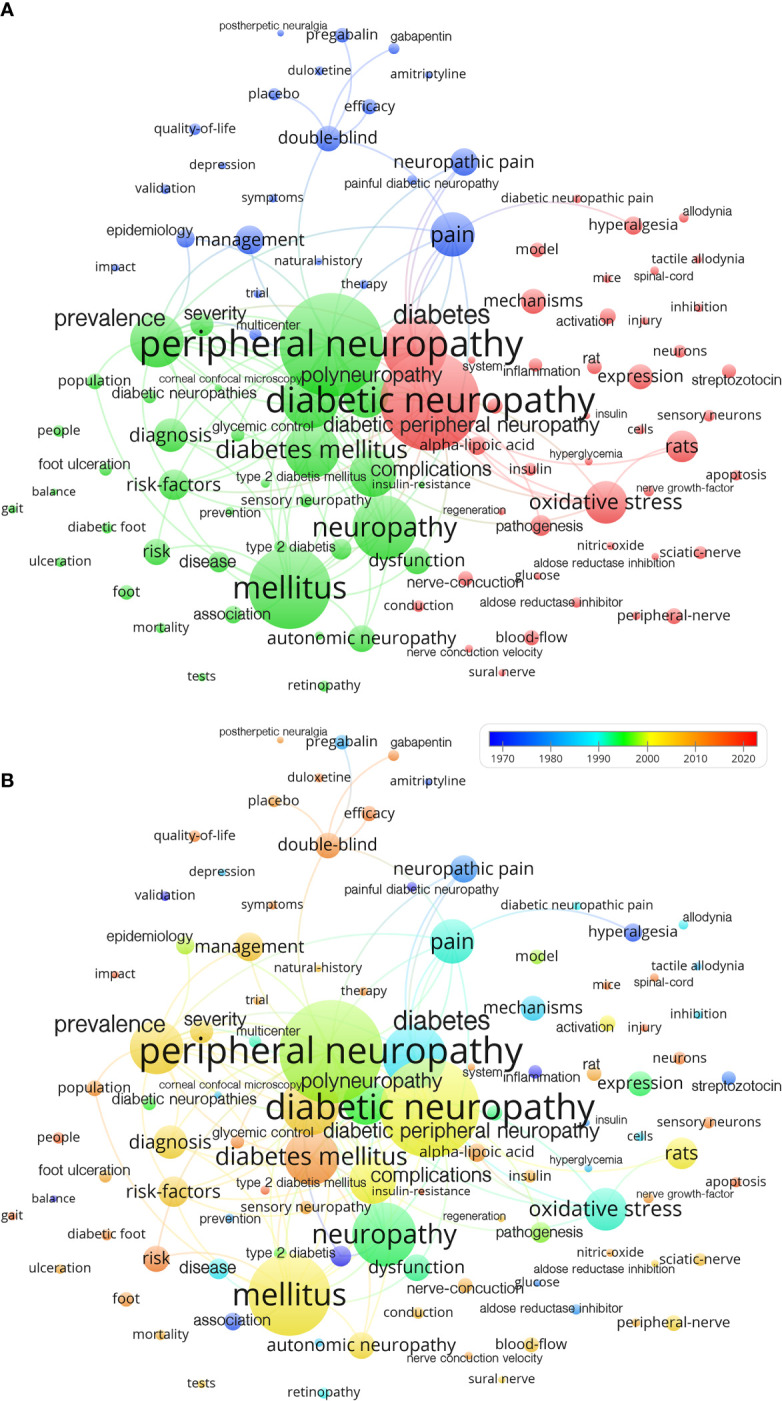
Main keyword analysis (occurrence: 10 times): **(A)** Cluster of keywords. **(B)** Time assignment of keywords 1970-2021.

Publications on diabetic neuropathy primarily focused on several key scientific themes that garnered significant attention. These included peripheral neuropathy, neuropathy, prevalence, diagnosis, risk factors, and pain. These core subjects were frequently explored and analyzed within the scientific literature pertaining to diabetic neuropathy, highlighting their vital importance in understanding the condition. Researchers and scholars extensively investigated these fundamental aspects to unravel the characteristics, prevalence rates, diagnostic methodologies, risk factors, and the intricate nature of pain associated with diabetic neuropathy. The Keywords cluster shows the focus of research in the field (**Figure 1A**).

An intriguing observation arises when examining the evolution of keywords over the past five decades (1970-2021) in the context of diabetic neuropathy. Notably, a noticeable shift towards clinical trials and translational research becomes apparent. This shift indicates a growing emphasis on conducting clinical trials and translating scientific findings into practical applications within the field of diabetic neuropathy. Over time, researchers have increasingly focused on investigating the efficacy of various interventions and therapies, aiming to bridge the gap between scientific discoveries and their implementation in clinical practice. This trend highlights the evolving landscape of research in diabetic neuropathy and the increasing importance placed on clinical applications and patient-oriented outcomes (**Figure 1B**).

### Publication development over time

The objective of this section was to evaluate the chronological progression of publications over time.

Our understanding of diabetic neuropathy has evolved significantly over time. In 1864, de Calvi proposed a hypothesis linking neurological dysfunction to diabetes mellitus, while Charcot provided a comprehensive clinical description in 1890. These contributions marked important milestones in the field. Since then, our knowledge of diabetic neuropathy has continued to expand through scientific research and clinical observations, driving advancements in prevention, diagnosis, and management. The insights provided by de Calvi and Charcot serve as a foundation for ongoing efforts to further unravel the complex mechanisms underlying diabetic neuropathy (12). In 1905, a notable publication by Williamson in Lancet presented a distinct paper reporting the absence of vibration perception in certain individuals with diabetes. This paper shed light on an important clinical observation regarding the sensory impairment associated with diabetes (13). An early significant contribution to the study of Diabetic Neuropathy was made by Minkowski with the publication titled “Opinions about a Case of Amyotrophic Lateral Sclerosis.” This article carries a historical importance as it enhances our comprehension of the association between diabetes and neuropathic disorders. Minkowski’s pioneering work in this domain has played a crucial role in advancing our knowledge of diabetic neuropathy, making notable contributions to the field of research in this area (14). In 1945, a noteworthy paper titled “Review of One Hundred Cases of Diabetic Neuropathy” was published by Rudy and Epstein. This paper presents an intriguing examination of a hundred cases, offering valuable insights into the characteristics and manifestations of diabetic neuropathy (15).

Out of a total of 5,411 articles identified on the topic of diabetic neuropathy from 1900 to 2021, a significant portion of 1,706 articles (31%) were published specifically between 2015 and 2021. This indicates a substantial increase in research output and attention given to the subject during this recent period.

**Figure 2:**
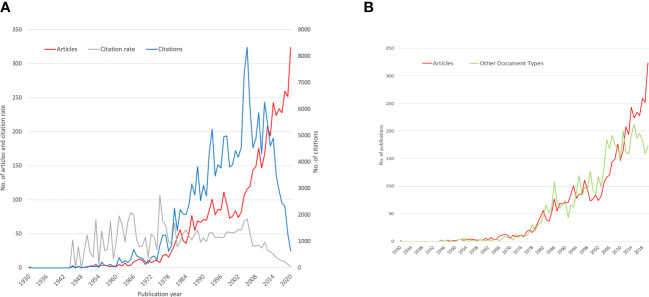
Chronological Development and Impact of Publications in Global Diabetic Neuropathy Research; **(A)** Output, Citations, and Average Annual Citation Rates; **(B)** Comparison number of Original Papers vs. Other Types of Publications in Global Diabetic Neuropathy Research.

The publication output related to diabetic neuropathy exhibited a gradual rise during the first four decades of the 20th century. However, it wasn’t until the late 1980s that the annual article count exceeded 100. Subsequently, from 2010 onwards, the number of annual articles identified surpassed 200. Similarly, the number of citations has shown a slow but steady increase since 1978, reflecting the growing recognition and impact of research on diabetic neuropathy (**Figure 2A**)

**Figure 2B** represents the development of publication parameters (original articles and reviews) from 1930 to 2020. The graph presents the number of articles, number of citations, and average citation rate per year in the field of Diabetic neuropathy.

### Diabetic neuropathy research area analysis

**Figure 3:**
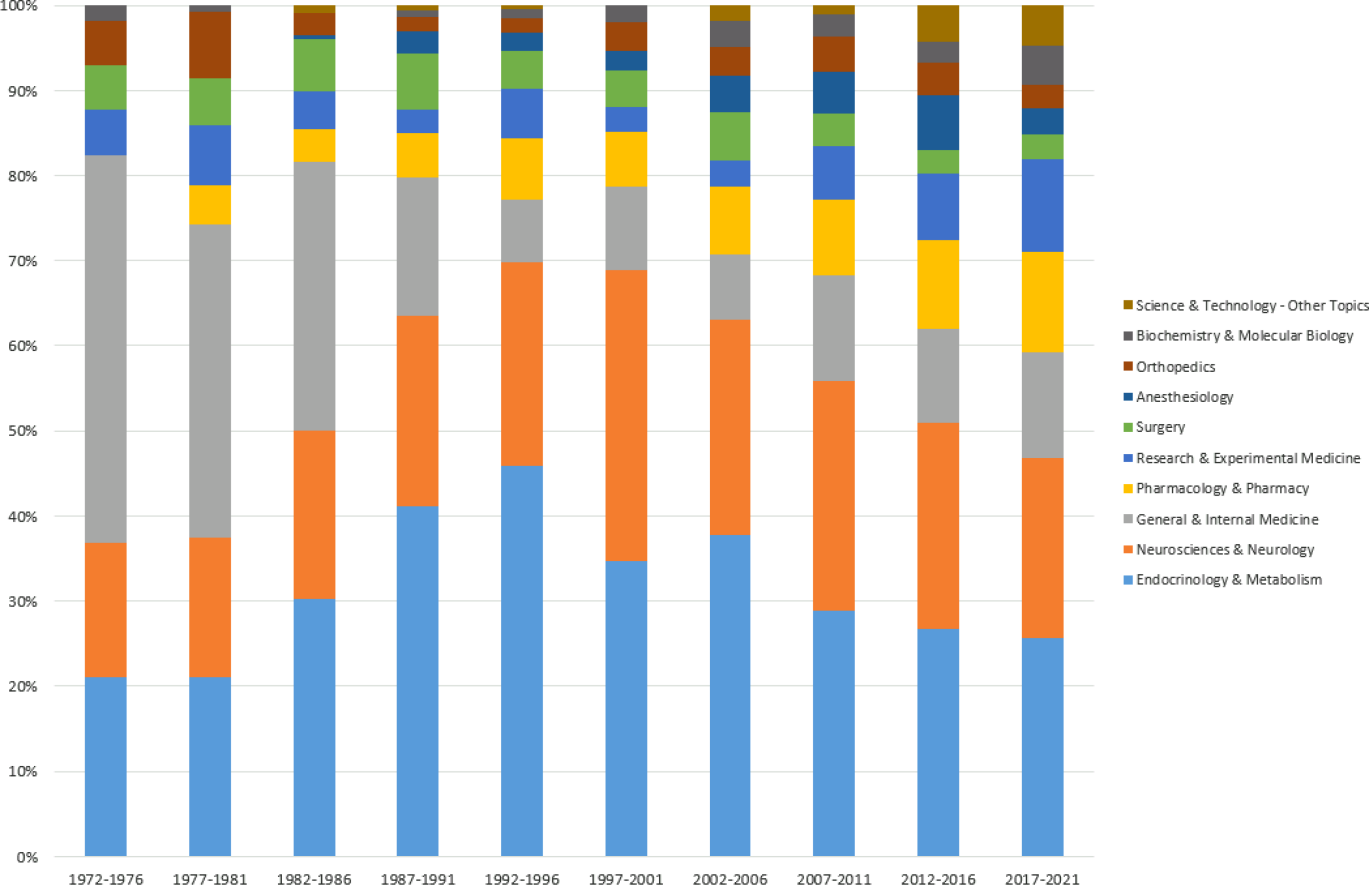
Analyzing Subject Areas in Diabetic Neuropathy Research: A Comprehensive Evaluation from 1970 to 2021. (Relative Proportions of Assigned Subjects in 5-Year Intervals from 1972 to 2021).

The analysis of diabetic neuropathy research and publications indicates a notable shift in focus across various medical disciplines over different time periods. Specifically, between 1970 and 1990, the majority of publications centered around “General and Internal Medicine.” This trend changed between 1987 and 2006, as the emphasis shifted towards “Endocrinology and Metabolism.” Notably, from 2006 to 2021, the predominant focus of research in the field of diabetic neuropathy has been “Neuroscience and Neurology.” To better visualize this shift, **Figure 3** illustrates the distribution of the top ten medical areas and their respective percentages over the years.

In the country-specific analyses conducted on the field of diabetic neuropathy between 1970 and 2021, a total of 94 countries or regional territories were identified from which authors published articles. This highlights the global participation and contribution of researchers across various geographical locations in advancing the knowledge and understanding of diabetic neuropathy.

**Figure 4:**
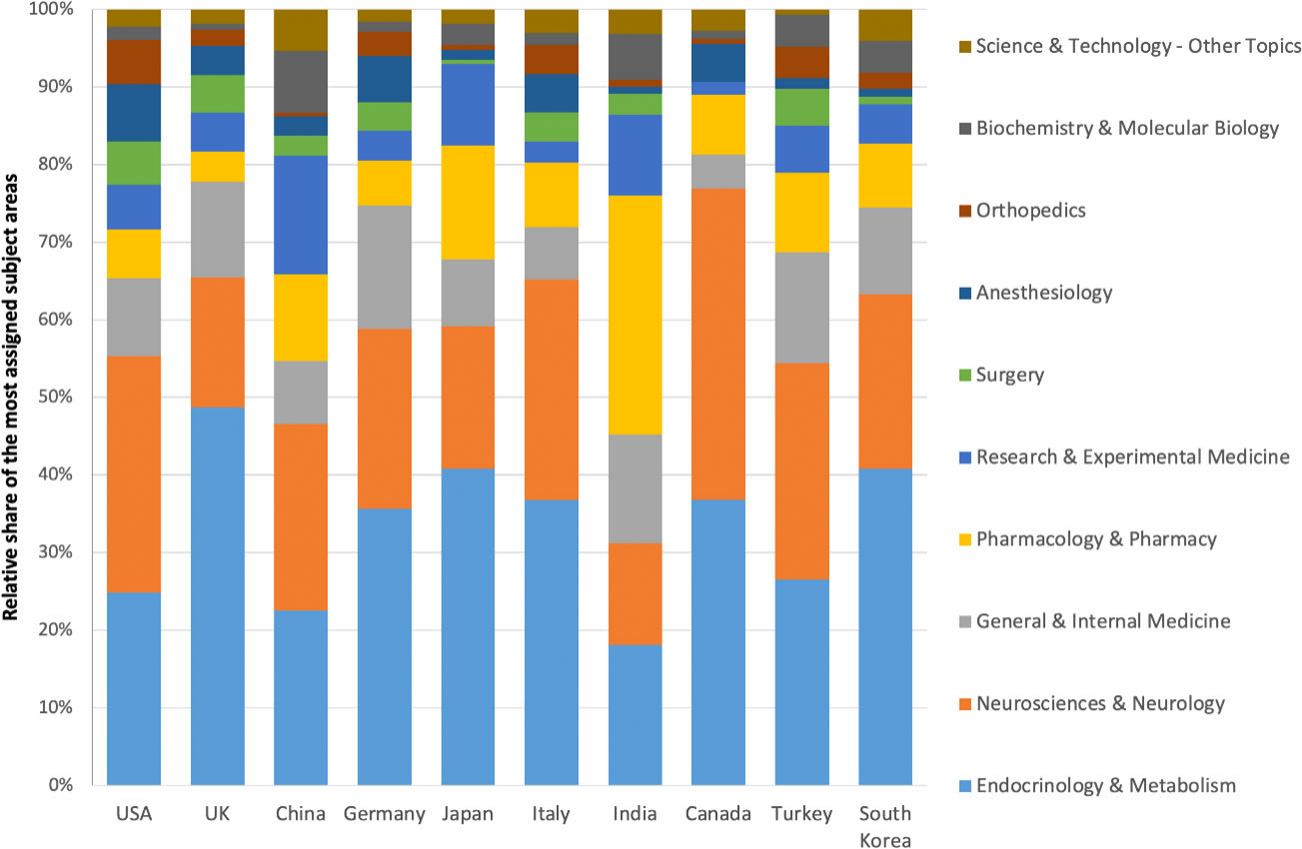
Relative Share of Most Assigned Subject Areas in the Top Ten Research-Active Countries.

Out of the total number of articles analyzed, 5,244 articles (96.91% of the sample) could be attributed to a specific country of origin. The analysis revealed that authors affiliated with the United States of America (USA) exhibited the highest productivity, contributing the largest number of diabetic neuropathy-related papers (n=1,296). The United Kingdom (UK) ranked second with 630 articles, followed by China (n=471), Germany (n=401), and Japan (n=370). These countries emerged as the leading contributors in the field of diabetic neuropathy research based on the analyzed dataset (**Figure 4**, **Table 1**).

**Table 1:** Percentage of the top 15 countries with top subject areas and the most published articles in the field of diabetic neuropathy.

Country	Articles	Endocrinology & Metabolism	Neurosciences & Neurology	General & Internal Medicine	Pharmacology & Pharmacy
USA	1296	0.26	0.30	0.085	0.06
UK	630	0.48	0.16	0.10	0.03
China	471	0.20	0.22	0.067	0.09
Germany	401	0.37	0.20	0.14	0.05
Japan	370	0.40	0.17	0.072	0.14
Italy	300	0.35	0.24	0.067	0.09
India	227	0.17	0.12	0.12	0.28
Canada	185	0.35	0.40	0.04	0.08
Turkey	160	0.24	0.24	0.12	0.09
Netherlands	146	0.30	0.30	0.02	0.06
South Korea	133	0.27	0.16	0.08	0.06
France	123	0.42	0.22	0.09	0.04
Iran	112	0.26	0.11	0.09	0.18
Denmark	104	0.36	0.30	0.03	0.11
Brazil	101	0.20	0.182	0.03	0.02

Upon conducting a country-specific analysis of subject area activities for the top ten most active countries, it became evident that researchers have been increasingly focusing on “Endocrine and Metabolism” as well as “Neuroscience and Neurology.” These areas have gained prominence in their scientific endeavors. Notably, in China, there is a significant emphasis on “Research and Experimental Research,” indicating a strong commitment to experimental studies in the field of diabetic neuropathy. On the other hand, in India, researchers have shown a notable focus on “Pharmacology and Pharmacy,” highlighting their interest in exploring therapeutic approaches and pharmacological interventions for diabetic neuropathy (**Figure 4**).

### Density equalizing mapping projections

**Figure 5:**
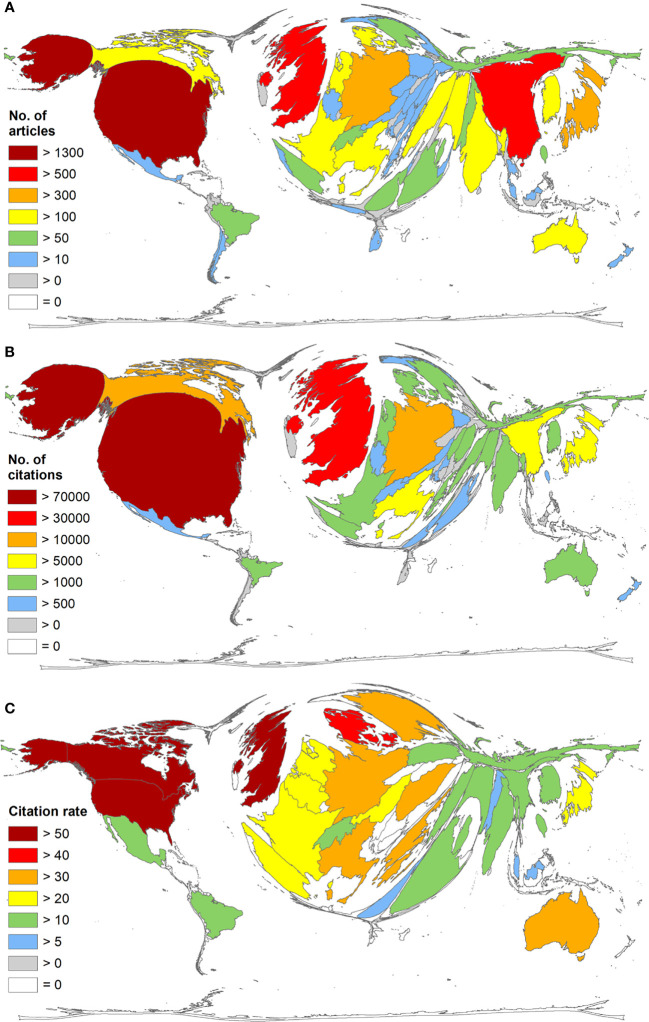
Global publication output on Diabetic Neuropathy from 1930-2021. **(A)** The number of articles per country. **(B)** number of Citations **(C)** citation rate (threshold = 30 publications on DN). Density equalizing, colors and territorial sizes indicate the numbers of related publications, citations and citation rate per country.

The Density-Equalizing Mapping Projections (DEMP) analysis uncovered a distinctive distortion of the world map, indicating a significant concentration of publication numbers in Northern America (n > 1000) and Western Europe (n > 500) (**Figure 5A**). A similar pattern can be observed when analyzing the number of citations (**Figure 5B**) and citation rates, mirroring the concentration of research impact in Northern America and Western Europe (**Figure 5C**).

The top three countries in terms of article numbers (n) were the USA (n = 1337), UK (n = 644), and China (n = 510). When considering citation numbers (c), the top three countries were the USA (c = 71,681), UK (c = 35,281), and Germany (c = 13,389). Notably, China ranked 7th in terms of citation numbers (c = 5173). 

The three leading countries in terms of average citation rate (cr) were Canada (cr = 56.44), UK (cr = 54.78), and USA (cr = 53.61). However, China ranked 60th in terms of average citation rate (cr = 10.14), indicating a relatively lower average impact of the research articles from China compared to the leading countries. Furthermore, **Figure 5** presents the Global publication output on Diabetic Neuropathy from 1930-2021 and shows (a) the number of articles per country (b) number of Citations (c) citation rate.

**Table 2** lists the top 10 articles that received the most citations until the date of evaluation.

**Table 2:** Top 10 cited articles in the field of Diabetic Neuropathy.

Country	Authors	Year	Journal	Citations	Title
United Kingdom, United States, Canada, Germany	Boulton AJM, et al. (16)	2005	Diabetes Care	1134	Diabetic neuropathies: a statement by the American Diabetes Association
United Kingdom	Young MJ et al. (17)	1993	Diabetologia	963	A multicentre study of the prevalence of diabetic peripheral neuropathy in the United Kingdom hospital clinic population
United States	Dyck PJ, et al. (18)	1993	Neurology	954	The prevalence by staged severity of various types of diabetic neuropathy, retinopathy, and nephropathy in a population-based cohort: the Rochester Diabetic Neuropathy Study
United States	Max MB, et al. (19)	1992	N Engl J Med.	798	Effects of desipramine, amitriptyline, and fluoxetine on pain in diabetic neuropathy
United Kingdom, Greece, Romania	Tesfaye S, et al. (20)	2005	N Engl J Med.	756	Vascular risk factors and diabetic neuropathy
United States, United Kingdom, Italy	Feldman EL, et al. (21)	1994	Diabetes Care	744	A Practical Two-Step Quantitative Clinical and Electrophysiological Assessment for the Diagnosis and Staging of Diabetic Neuropathy
United Kingdom	Ewing DJ. et al. (22)	1980	Q J Med.	660	The natural history of diabetic autonomic neuropathy
United States	Goldstein DJ. et al. (23)	2005	Pain	524	Duloxetine vs. placebo in patients with painful diabetic neuropathy
United States	Frampton JE. et al. (24)	2004	Drugs	487	Pregabalin: in the treatment of painful diabetic peripheral neuropathy
United States	Max MB, et al. (25)	1987	Neurology	484	Amitriptyline relieves diabetic neuropathy pain in patients with normal or depressed mood

### Socio-economic analysis of diabetic neuropathy publishing

**Figure 6:**
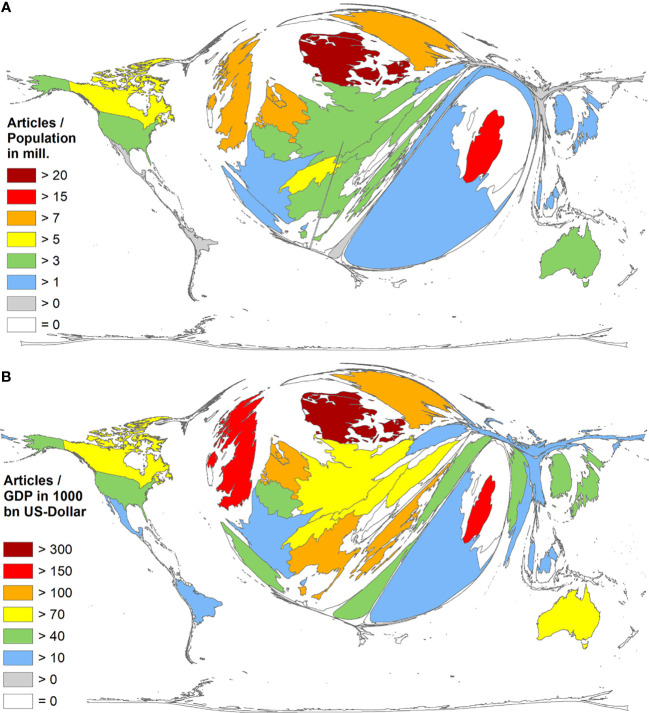
Density Equalizing Map of the socio-economic parameters of the publishing countries with ≥30 articles. **(A)** Ratio of number of articles and population (in mill.). **(B)** Ratio of number of articles and gross domestic product in 1,000 bn USD.

First, the analysis of country-specific diabetic neuropathy publications was conducted in relation to the population size measured in million inhabitants (R_POP_). This approach aimed to examine the association between the number of publications in each country and the corresponding population size (**Figure 6**). By considering the population size as a factor, the study sought to provide a comprehensive understanding of the distribution and representation of diabetic neuropathy research across different countries, accounting for variations in population size. According to the data presented in **Table 2**, Denmark emerged as the most active high-income country in terms of research productivity, with a Relative Publication Output per capita (R_POP_) of 20.27 publications per inhabitant. Qatar followed closely with an R_POP_ of 18.01, while the United Kingdom (UK) ranked third with an R_POP_ of 9.54. The Netherlands secured the fourth position with an R_POP_ of 7.95, and Sweden rounded off the top five with an R_POP_ of 7.87.

In order to assess the economic strength of countries in relation to their population and its impact on diabetic neuropathy research output, the gross domestic product (GDP) was utilized as a marker. The countries’ GDP, measured in 1,000 billion (bn) USD, served as an indicator of their total economic strength. This parameter was then linked to the country-specific diabetic neuropathy publications to create the ratio RGDP. By employing this approach, the study aimed to investigate the relationship between a country’s economic resources and its research productivity in the field of diabetic neuropathy. **Table 3** displays the rankings of the top 32 countries based on the evaluations conducted.

**Table 3:** Evaluation of Countries’ ranking (1970-2021) according to the socioeconomic parameters for diabetic neuropathy research of the most active countries (UIS.stat database (UNESCO)) (9, 26), sorted by R_POP_.

Country	Articles	Population in mill	GDP in 1000 bn PPP	RPOP	Rank POP	RGDP	Rank GDP
Denmark	117	5.77	0.35	20.27	HI 1	336.09	HI 1
Qatar	51	2.83	0.27	18.01	HI 2	186.63	HI 3
United Kingdom	644	67.53	3.26	9.54	HI 3	197.82	HI 2
Netherlands	136	17.10	1.03	7.95	HI 4	131.46	HI 6
Sweden	79	10.04	0.57	7.87	HI 5	137.61	HI5
Switzerland	53	8.59	0.61	6.17	HI 6	87.07	HI 10
Singapore	30	5.76	0.58	5.21	HI 7	51.88	HI 18
Canada	188	37.41	1.93	5.03	HI 8	97.41	HI 8
Greece	50	10.47	0.34	4.77	HI 9	148.59	HI 4
Germany	388	83.52	4.66	4.65	HI 10	83.27	HI 11
Italy	279	60.55	2.66	4.61	HI 11	104.69	HI 7
Austria	41	8.96	0.52	4.58	HI 12	78.14	HI 13
Australia	107	25.20	1.35	4.25	HI 13	79.12	HI 12
United States	1337	328.24	21.37	4.07	HI 14	62.55	HI 14
Hungary	32	9.69	0.33	3.30	HI 15	96.39	HI 9
Belgium	37	11.54	0.63	3.21	HI 16	59.07	HI 17
Japan	341	126.25	5.46	2.70	HI 17	62.46	HI 15
South Korea	136	51.23	2.22	2.65	HI 18	61.12	HI 16
Spain	99	46.74	1.99	2.12	HI 19	49.82	HI 19
France	130	64.99	3.32	2.00	HI 20	39.21	HI 20
Turkey	160	83.43	2.33	1.92	UMI 1	68.80	UMI 1
Saudi Arabia	54	34.27	1.68	1.58	HI 21	32.22	HI 22
Iran	119	82.91	0.00	1.44	LMI 1	0.00	LMI 4
Poland	48	37.89	1.30	1.27	HI 22	36.94	HI 21
Malaysia	33	31.95	0.94	1.03	UMI 2	34.98	UMI 2
Egypt	77	100.39	1.23	0.77	LMI 2	62.61	LMI 1
Russia	76	145.87	4.28	0.52	UM13	17.75	UMI 5
Brazil	98	211.05	3.22	0.46	UMI	30.43	UMI 3
China	510	1433.78	23.46	0.36	UM15	21.74	UMI 4
Pakistan	53	216.57	1.06	0.24	LM13	50.10	LMI 2
Mexico	31	127.58	2.60	0.24	UMI	11.91	UMI 6
India	237	1366.42	9.61	0.17	LMI4	24.66	LMI 3

Sources for GDP (Current prices in 1,000 bn US Dollars) and GDP per capita (current prices in 1,000 US Dollars): IMF. HI, high-income country; UMI, upper middle-income country; LMI, lower-middle-income countries.

### Network analysis of international diabetic neuropathy research

**Figure 7:**
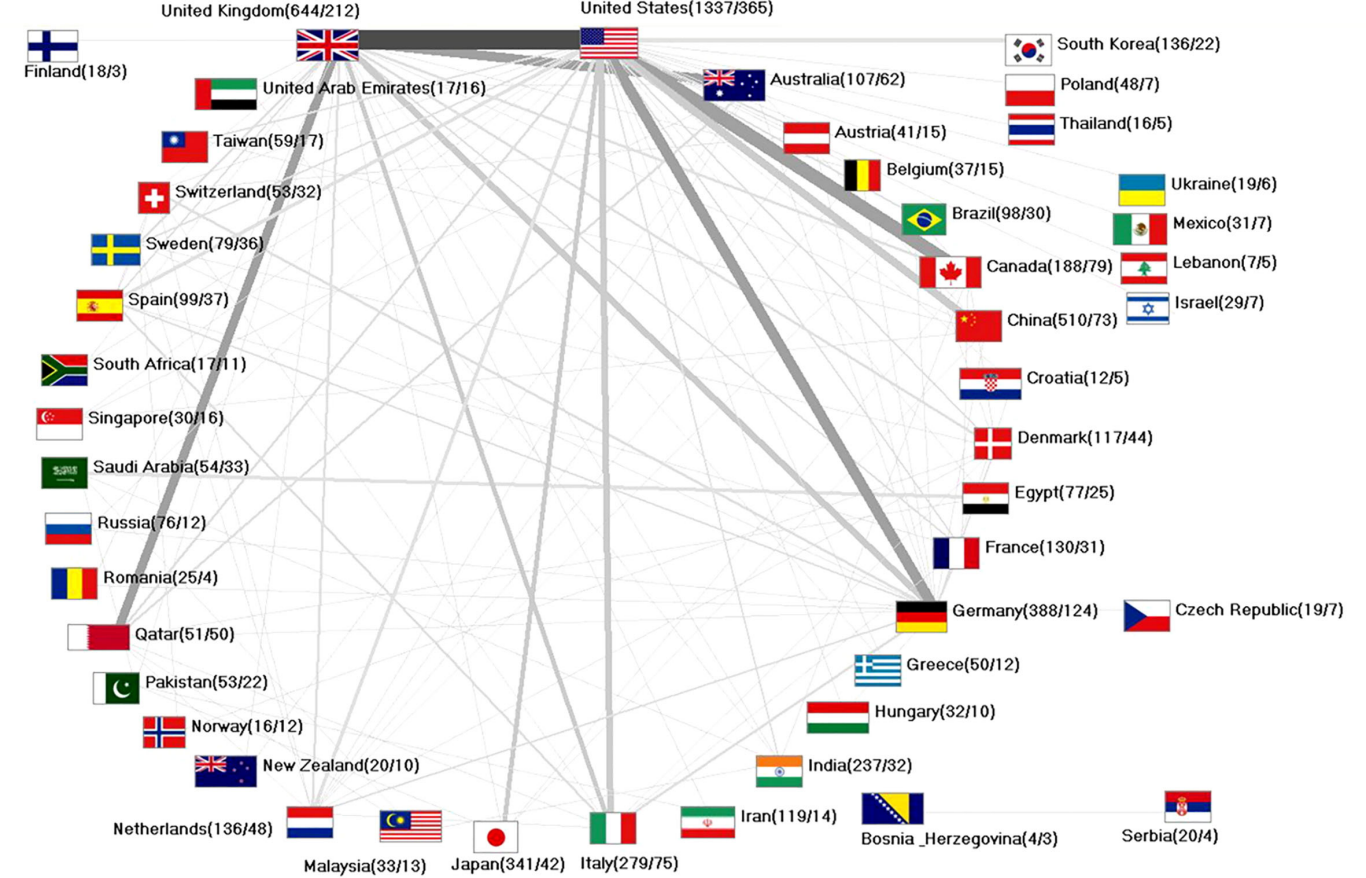
Global network of international collaborations on Diabetic Neuropathy. Collaborating countries with ≥ 10 joint bilateral articles (display threshold). Values in brackets: number of articles/number of collaborating articles. The thickness of the bars corresponds to the number of bilateral collaborations between linked countries.

A total of 762 international collaborations, accounting for 14.08% of all articles, were identified in the study. Among these collaborations, US-American authors were involved in 365 collaborative articles with researchers from other countries. This was followed by cooperation (number of collaborative articles = n_coop_); researchers based in the UK participated (n_coop_= 212). The most prominent collaboration occurred between the USA and the UK, accounting for 83 collaborative articles. This was followed by collaborations between the USA and Canada (n_coop_ = 56), USA and Germany (n_coop_ = 47), UK and Qatar (n_coop_ = 42), and UK and Australia (n_coop_ = 41), as illustrated in **Figure 7**.

## Discussion

Diabetic neuropathy (DN) is a prevalent and expensive chronic microvascular complication that affects individuals with type 1 and type 2 diabetes, including young people. It can result in severe consequences such as foot ulceration and amputation. DN also impacts a significant number of individuals worldwide who may have undiagnosed diabetes, prediabetes, or obesity. Timely detection and intervention are crucial in order to prevent these complications. Alarmingly, the global number of diabetes-related amputations has been rising, highlighting the widening disparities and equity concerns in diabetes care (27). Therefore, it is imperative to acquire a comprehensive understanding of the disease, including its pathogenesis and mechanisms, across various patient populations. This necessitates precise study designs and large-scale randomized clinical trials. These rigorous investigations are essential to identify effective interventions that can prevent the onset of diabetic neuropathy, facilitate early diagnosis, and ultimately achieve optimal therapeutic outcomes for patients.

The primary objective of the present study was to conduct a comprehensive analysis of the global research landscape in diabetic neuropathy (DN). The study aimed to offer valuable insights to scientists, funders, and decision-makers regarding the current status of research in DN as well as future directions. By providing a holistic overview of the research landscape, this analysis aimed to assist stakeholders in making informed decisions and advancing research efforts in the field of DN.

A total of 5,244 articles related to diabetic neuropathy, published between 1900 and 2021, were identified using specific search terms. To ensure the inclusion of rigorous scientific studies, the analysis focused solely on original articles, excluding reviews, letters, and other potentially non-peer-reviewed publications. The data acquisition was carried out using the Web of Science (WoS) database. WoS was chosen for its reputation of curating high-quality publications through a rigorous selection process, thereby guaranteeing a high level of scientific integrity in the included articles.

Since 1900, the production of articles on diabetic neuropathy has demonstrated a consistent upward trend. However, a significant disparity in research activity across geographical regions has been observed. The majority of scientific contributions originate from countries situated in the northern hemisphere, particularly within North America and Europe. In contrast, countries in Latin America and Africa, characterized by higher disease prevalence, mortality rates, and limited access to advanced healthcare, have not prominently featured as major research powerhouses in this field. Consequently, productive research collaborations involving these regions have been relatively scarce. This discrepancy underscores the need to address the imbalance in research activity and foster greater inclusivity in scientific collaborations, ensuring the involvement of diverse regions to advance our understanding and management of diabetic neuropathy on a global scale.

The identified need to involve non-high-income countries in scientific collaborations in the field of diabetic neuropathy emphasizes an opportunity for the exchange of ideas, knowledge, and epidemiological data, ultimately benefiting all parties involved. The unique strength of this study lies in its ability to showcase the global scientific productivity in diabetic neuropathy research over a span of 120 years. By recognizing and addressing the existing gaps in research participation, this study highlights the potential for fostering mutually beneficial collaborations that can contribute to advancements in understanding, prevention, and treatment of diabetic neuropathy on a global scale.

Several limitations should be acknowledged in this study. Firstly, it is important to recognize that the search methodology employed may not capture all articles published on diabetic neuropathy since 1900. The utilization of the WoS database, which primarily focuses on English-language publications, may introduce a language bias, potentially resulting in an underrepresentation of non-English literature. However, it is worth noting that English is widely regarded as the language of science, and many non-native English speakers choose to publish their high-quality scientific work in English-language journals. Therefore, while some non-English literature may be overlooked, the impact of this bias is expected to be minimal.

Additionally, the WoS database presents publications specifically related to the chosen search terms, and it is possible that relevant articles may have been missed due to variations in terminology or indexing inconsistencies. Despite these limitations, the study provides valuable insights into the global scientific productivity on diabetic neuropathy over the examined 120-year period.

It is important for future studies to consider alternative databases, language sources, and search strategies to further enhance the comprehensiveness and inclusivity of research analysis in the field of diabetic neuropathy.

Furthermore, we acknowledge that there are other platforms available, such as EMBASE or Google Scholar, which catalogue journal articles in the medical field. These platforms may potentially identify a different set of articles related to the search terms. However, it is important to note that these alternative platforms cover different time periods and may exhibit varying levels of accuracy in citation analysis. Additionally, they may lack certain unique tools and features offered by the Web of Science (WoS), such as the Journal Citation Reports. Considering these factors, we made the decision to refrain from utilizing these platforms in our study, opting to focus specifically on the WoS database to maintain consistency and take advantage of its comprehensive coverage and robust citation metrics (28). In addition, we acknowledge that attempting to assess scientific quality based on citation parameters can be challenging. Citations serve as a measure of recognition within the research community and can be influenced by phenomena such as the Matthew effect or self-citations. These factors may introduce biases and potentially impact the accuracy of evaluating scientific quality solely based on citation metrics. While citation analysis provides valuable insights into the impact and visibility of research, it should be interpreted with caution, considering the potential limitations and biases associated with this approach. Therefore, a comprehensive evaluation of scientific quality should involve multiple indicators and considerations, including rigorous peer review, expert assessments, and other qualitative measures (29).

Lastly, to account for economic opportunities in research and related infrastructure, a ratio of country-specific research output since 1900 to the GDP in 2021 was calculated. It is important to acknowledge that the GDP of certain countries has undergone substantial changes in recent decades. However, considering the positive association between GDP and scientific performance in both developed and developing countries, we deem the impact of these GDP fluctuations over time to be insignificant in terms of the scientific value of the analysis. By incorporating the GDP ratio, the study aimed to provide insights into the relationship between economic resources and research output in the field of diabetic neuropathy, acknowledging that economic factors can influence research capabilities and productivity to a certain extent (30).

## Conclusion

As demonstrated in this study, there has been a substantial increase in the number of basic, pre-clinical, and clinical studies conducted in the field of diabetic neuropathy over the years. This finding indicates a growing interest and investment in understanding and addressing the complexities of diabetic neuropathy. The expanding body of research signifies the scientific community’s recognition of the importance of advancing knowledge in this area and highlights the dedication to finding effective solutions for the prevention, diagnosis, and treatment of diabetic neuropathy.

Nevertheless, despite advancements in the current standards of clinical care, it is disheartening to acknowledge that a significant number of patients continue to endure the devastating consequences of diabetic neuropathy. Despite ongoing efforts to improve prevention, diagnosis, and treatment, this complication of diabetes continues to inflict suffering on millions of individuals. The persistence of diabetic neuropathy highlights the urgent need for further research and innovative approaches to alleviate the burden and enhance the quality of life for those affected.

Undoubtedly, research, particularly clinical research, holds immense potential to make a positive impact on patient outcomes. By improving and expanding research efforts, we can enhance access to effective treatments and interventions, ultimately reducing uncertainty and improving patient outcomes. The generation of robust evidence through clinical research plays a pivotal role in informing decision-making processes, guiding the approval and adoption of new treatments within healthcare systems. This collaborative effort fosters a more effective and efficient healthcare landscape, benefiting individuals and communities worldwide (31).

In light of the global challenges we have faced in recent years, such as climate change, the COVID-19 pandemic, and healthcare inequalities, it is clear that addressing the complications of diabetic neuropathy requires a collaborative approach on a global scale. The complexity and magnitude of these challenges necessitate the collective efforts and collaboration of various stakeholders, including researchers, healthcare professionals, policymakers, funding bodies, and governments worldwide. While the scope of these challenges may appear daunting, it is essential to maintain a sense of ambition and determination in our pursuit of solutions. The interconnected nature of these global issues underscores the need for comprehensive and integrated approaches that transcend geographical boundaries. Through fostering global research collaboration, we can leverage the collective expertise, resources, and perspectives of diverse stakeholders to develop innovative solutions, improve healthcare outcomes, and alleviate the burden of diabetic neuropathy. Collaboration should not be seen as an excuse for complacency, but rather as an opportunity to harness the strengths and capabilities of different stakeholders and drive meaningful change at a global level.

It is crucial to prioritize scientific research aimed at enhancing our understanding of diabetic neuropathy (DN) and its underlying pathophysiology, with a clear focus on potential clinical implications. Future research should prioritize prevention, precision medicine, and the development of improved biomarkers and treatment strategies. Despite successful strategies, many preclinical and clinical studies, as well as clinical drug development, face high failure rates. Addressing these challenges is essential for progress in DN research. By investing in rigorous scientific endeavors, we can improve diagnostics and advance therapeutic interventions, ultimately improving the lives of those affected by DN (32). The current state of research may overlook certain aspects of target validation and drug optimization, as well as the absence of sensitive biomarkers. To address these challenges, it is crucial to enhance our systematic analysis of the evidence at each stage of translation. By prioritizing biomarkers with the strongest evidence, we can identify the most promising candidates that have the potential to yield successful outcomes. This strategic approach will enable us to optimize the translation of research findings and improve the development of effective interventions in the long run (33).

Over the past decade, funding for research, including research grants, capital investments, and overall funding, in the field of diabetic neuropathy has not experienced a substantial increase compared to other areas of diabetes research and prevalent chronic diseases such as dementia, cancer, and cardiovascular disease. This disparity in funding allocation highlights the pressing need for enhanced support from funding organizations and policymakers. It is imperative for these entities to acknowledge the urgency and gravity of this widespread complication of diabetes and prioritize research funding accordingly. By investing in comprehensive and robust research endeavors, we can advance our understanding of diabetic neuropathy, develop effective interventions, and alleviate the burden on individuals affected by this condition. Sufficient research funding is of paramount importance in addressing the challenges associated with diabetic neuropathy and achieving significant advancements in patient outcomes and overall quality of life.

In the current paper, while we thoroughly explored the influence of GDP and other economic factors, we recognize that we did not delve extensively into the level of investments, infrastructure, and funding from various sources such as governments, patients, research charities, and the pharmaceutical industry. Understanding the financial support and resources allocated to diabetic neuropathy research is crucial for comprehending the overall landscape and potential impact of the field. Government funding, contributions from patients and advocacy groups, as well as investments from research charities and the pharmaceutical industry, all play pivotal roles in driving progress in this area. By incorporating a more comprehensive analysis of these financial aspects in future studies, we can gain a deeper understanding of the resources available for research and potentially identify areas where increased investments and collaborations could further advance diabetic neuropathy research. Such efforts are vital to address the growing global burden of diabetic neuropathy and work towards improved treatments and outcomes for affected individuals.

In light of the global increase in cases of diabetic neuropathy, there is an urgent need to discover improved treatments and more effective management strategies. This urgency highlights the importance of enhancing the infrastructure for diabetic neuropathy research. It is crucial to recognize that this need is particularly significant for countries with low-income economies, considering their low participation in research efforts.

By expanding our understanding of the factors that influence research outcomes and taking into account the level of investments, infrastructure, and funding, we can work towards fostering advancements in diabetic neuropathy research and ultimately improve patient care and outcomes.

In conclusion, this study’s outcomes may have implications for shaping future research priorities and funding initiatives, directing resources toward addressing the identified knowledge gaps and promoting innovative approaches to personalized care. Ultimately, the goal is to alleviate the burden of DN, improve patient outcomes, and enhance the overall quality of life for individuals affected by this debilitating condition.

## Data availability statement

The bibliometric data are the property of the Web of Science database and were obtained from it. Therefore, the authors are not allowed to pass on the data publicly or privately. Any researcher with access to the Web of Science database can obtain the data using the methods described in the paper. Readers who do not have access to Web of Science should contact Clarivate Analytics to obtain a license.

## Author contributions

The study was initiated by MT, DK, and DG. DK conducted the data processing, while DK and MT performed the data analysis. MT and DK engaged in discussions regarding the data and carried out analysis during the review process and writing the paper. MT, DK, HF, and DG contributed to the writing and reviewing of the paper. All authors have thoroughly read and approved the final version of the manuscript.
